# Supplementary material for: Demographic and socio-economic predictors of physical activity among young adults in Lephalale, Limpopo Province, South Africa
Source: PLOS Glob Public Health. 2026 Feb 23;6(2):e0006003. doi: 10.1371/journal.pgph.0006003 (PMC12928425; doi:10.1371/journal.pgph.0006003)
Supplement: Attachment — Submitted filename: Reviewer comments_03102025_final.pdf [file pgph.0006003.s002.pdf]

Review date October 02, 2025

Journal PLOS Global Public Health

Manuscript ID PGPH-D-25-01139

Exploring the interplay between socioeconomic and demographic factors in shaping physical activity behaviours among young adults in Elisrus

Reviewer's report

In summary, the authors reported that the daily PAs among young men and women were 15% and 10%, respectively, and the weekly PAs among young females (35%) were slightly greater than those among young males (30%). Two main predictors of PA among young adults aged 18--29 years in the rural area of Elisrus were those with higher education at the university/college level and those working full-time jobs.

Reviewing the manuscript, I have some questions and comments that authors need to further address and clarify throughout the manuscript before it co.uld be considered for publication PLOS Global Public Health journal. Here are the questions, comments and clarifications throughout the manuscript that could be addressed or revised to improve the clarity and content of the manuscript.

General comment: Please stick to a consistent name either *Elistrus, Ellistras or Lephalale* and use it along with the text. Avoiding switching the terms. It could confuse the readers.

Abstract

Discussion in the Abstract talking about gender disparities: I do not agree with the statement that *gender disparities in PA levels were observed, although they were not statistically significant in adjusted analyses*. In your final multivariate logistic regression, only higher education and full-time jobs were the two main predictors of PA among these young rural adults. Please readdress this properly.

Introduction

1- Second paragraph: The authors should detail few sentences specifically detail PA among young adults in few countries in Asia, Europe and Africa to get a better picture and patterns of PAs among youths in different geographical context with the support by citing sources.

Methods

2- … *"Geographically, it lies between latitudes 23°30' and 24°00' South and longitudes 27°30' and 28°00' East. The municipality borders four others— Blouberg, Modimolle, Mogalakwenai, and* Thabazimbi—should be removed. Readers may be not interested in this.

3- Third paragraph of the **Study setting** *"The local economy is predominantly driven … disproportionately affect rural communities in Lephalale"* should be shortened by half since it contained more than enough information to read.

4- In the **Study population,** the authors mentioned that *a total of 1063 participants were recruited*. However, in the abstract, *data from a community-based survey of 762 participants aged 18–29 years* were analysed. Please clarify this inconsistency.

5- In the **Data collection:** Please detail further whether the questionnaire was translated into a local language or in English, how the interview was conducted? Had the interviewers been trained to collect the data?

6- In the **Data analysis**: Definition of outcome variable (PAs) should be clearly stated. How were *physical activity levels (≥150 minutes of moderate-intensity activity per week)* based on what questions? Was it combined different questions?

7- Any ethical approval by University of University of Limpopo where authors were working?

Results

8- N = XXX should be specified in Table 1-4 so that readers can see the total sample in each table. It looked the N were not the same across the tables.

Discussion

9- **First paragraph**, the main finding summary of the study should reflect how low, moderate, or high PA across socioeconomic and demographic characteristics (most likely, if daily and weekly PA are added, it ranges between 45% and 55%), and only higher education and full-time jobs are the only two main predictors of PA. Please recheck it to reflect the main study finding.

10- **Third paragraph**, the authors stated that *sex differences in PA were also evident, with males engaging in more daily PA than females did, although females presented slightly higher rates of weekly engagement*. However, as shown in Table 4, there was no difference between female young adults and male young adults in terms of PAs (AOR = 0.85, 95% CI: 0.60--1.20, p value = 0.35). Please readdress this paragraph.

11- **Fourth paragraph** the authors stated that *in unadjusted analyses, higher income was associated with a significantly greater likelihood of meeting PA guidelines, while lower income correlated with a reduced likelihood*. However, as shown in Table 4, the results of multivariate logistic regression after controlling for other factors revealed that higher income was no longer independently associated with PA, with an AOR = 1.20 (95% CI: 0.90–1.50, p value = 0.25). Please readdress this paragraph.

I declare that I have no conflicting interest in reviewing this manuscript.
