# Supplementary material for: Demographic and socio-economic predictors of physical activity among young adults in Lephalale, Limpopo Province, South Africa
Source: PLOS Glob Public Health. 2026 Feb 23;6(2):e0006003. doi: 10.1371/journal.pgph.0006003 (PMC12928425; doi:10.1371/journal.pgph.0006003)
Supplement: Attachment — Submitted filename: Response to Reviewers.docx [file pgph.0006003.s003.docx]

**RESPONSE TO REVIEWERS**

1. **Reviewer**: In paragraph 1 of the introduction, The (Kwan....) citation should be deleted as it is not following the right guideline of the journal citation preferences.

**Authours**: Thank you for the comment. The (Kwan…) citation in paragraph 1 of the introduction has been removed to align with the journal’s citation guidelines.

2. **Reviewer**: In the last paragraph of the introduction, where is Lephalale? the province, the country should be added.

**Authours**: Thank you for the observation. In the last paragraph of the introduction, the location of Lephalale has been clarified to read “Lephalale, Limpopo Province, South Africa” to provide full geographic context.

3. **Reviewer**: In the last paragraph of the introduction, With this specific aims highlighted here, it does not fully reflect in the tittle of the study. The PA pattern and intensity does not align well with the title. If these are aims, the title need to be changed or rephrased.

**Authours:** Thank you for the valuable comment. The phrase “PA patterns based on frequency and intensity” in the last paragraph of the introduction has been revised for clarity and alignment with the study title. The text now reads: “…patterns of physical activity (PA) with respect to frequency and intensity…”. Additionally, the alignment between the study aims and the title has been reviewed, and appropriate revisions have been made to ensure consistency

4. **Reviewer**: In Study design and setting, this seems jumping here and there when it comes to determining the main objective/purpose of this study. This should be adequately looked into.

**Authours:** We appreciate the reviewer’s observation. The Study design and setting section has been revised to ensure a clearer and more logical flow. The main objective of the study is now explicitly stated at the beginning of the section, followed by a structured description of the geographical, demographic, and socio-economic characteristics of Lephalale. These contextual details are presented in a way that directly links them to the study’s focus on how socio-economic factors and age influence physical activity behaviours among young adults in rural settings.

5. **Reviewer**: Under the study population, red to black ink should be adopted. should black and white color.

**Authours:** We thank the reviewer for this observation. The suggested change has been implemented, the section on Study Population has been revised to adopt black ink, ensuring consistency and clarity in line with the recommendation.

6. **Reviewer**: Under the data collection, I am concerned about this, Since the survey used in this study was adapted from previous survey, what is the psychometric property of the newly adopted questionnaire? its reliability and validity of the new survey should be added. This could help those who wish to replicate the study elsewhere.

**Authours:**  Thank you for your valuable feedback regarding the psychometric properties of the adapted questionnaire. We recognize the importance of providing information on the reliability and validity of the survey instrument to support replication. The questionnaire used was adapted from the Global Physical Activity Questionnaire (GPAQ), which has well-documented reliability and validity across diverse populations. To ensure the adapted questionnaire was appropriate for the local context, a pilot study was conducted that demonstrated satisfactory internal consistency (Cronbach’s alpha > 0.7) and confirmed its reliability in measuring physical activity domains. Furthermore, the data for this study were obtained as secondary data from the Ellisras Longitudinal Study (ELS), which employs standardized and validated data collection protocols.

In response to your comment, we have now added these details regarding the psychometric properties and the source of the data in the Methods section under Data Collection to clarify the reliability and validity of the instrument.

7. **Reviewer**: Under the ethical clearance, does this study have ethical numbers? this should be added.

**Authours**: We thank the reviewer for this observation. The Ethical clearance section has been updated to include the relevant approval numbers as follows: “Ethical approval for the study was granted by the Turfloop Research Ethics Committee (MREC/P/204/2013: IR and TREC/323/2017: IR-Renewed).”

8. **Reviewer**: Under the discussion in the first paragraph, Please, see my comment about the aim of the study above.

**Authours**: We appreciate the reviewer’s feedback. The first paragraph of the Discussion has been revised to clearly reflect the main aim of the study as stated in the Introduction. It now reads: “This study investigated the demographic and socio-economic determinants of physical activity (PA) among young adults aged 18–29 years in Lephalale, Limpopo Province, South Africa. The primary objective was to examine how socio-economic status, age, and related demographic factors influence PA behaviours, thereby providing insights into context-specific determinants that could inform targeted public health interventions.”

9. **Reviewer**: I suggest the paper be read by experts in the field who is qualified to judge the scientific writing as I noticed that the work need to be rigorously edited for clarity.

**Authours:** We appreciate the reviewer’s observation regarding the clarity of the manuscript. In response, the paper has undergone a thorough language and technical review by a subject-matter expert with experience in scientific writing in the field of physical activity and public health. The revisions focused on improving sentence structure, refining terminology, and enhancing overall flow to ensure clarity and precision. We believe these changes have strengthened the readability and scientific quality of the manuscript.

10. **Reviewer**: The author need to make sure that the study follow STROBE checklist as it does not do in the current status of the paper.

**Authours:** We thank the reviewer for highlighting the importance of adhering to the STROBE (Strengthening the Reporting of Observational Studies in Epidemiology) checklist. The manuscript has been carefully reviewed against the STROBE guidelines, and all relevant items have now been addressed and incorporated. These include clearer articulation of the study design in the title and abstract, expanded details on participant selection and setting, specification of variables and data sources, clarification of bias minimisation measures, and comprehensive reporting of results with appropriate descriptive and inferential statistics. A completed STROBE checklist is now included as a supplementary file for the reviewer’s consideration.
