# Supplementary material for: Demographic and socio-economic predictors of physical activity among young adults in Lephalale, Limpopo Province, South Africa
Source: PLOS Glob Public Health. 2026 Feb 23;6(2):e0006003. doi: 10.1371/journal.pgph.0006003 (PMC12928425; doi:10.1371/journal.pgph.0006003)
Supplement: Attachment — Submitted filename: Rebuttal letter.docx [file pgph.0006003.s004.docx]

**REVIEWER #1**

**Comment 1:** The author should rephrase the title of the manuscript to read “Demographic and socio-economic predictors of PA among young adults in Elisrus.”

**Response:** We appreciate this suggestion and have revised the title to “Demographic and socio-economic predictors of physical activity among young adults in Lephalale.” We also ensured that “Lephalale” is used consistently throughout the manuscript to correctly reflect the study location. Revisions have been made on the title page and throughout the text.

**Comment 2**: The manuscript requires improvements in English language.

**Response:** Thank you for this helpful point. The entire manuscript has undergone careful language editing to improve clarity, grammar, flow, and overall readability.

**REVIEWER #2**

**Abstract**

**Comment 1**: The statement about gender disparities should be removed since results were not statistically significant after adjustment.

**Response:** Thank you for this important observation. We have removed any mention of gender differences from the Abstract and now highlight only variables that were statistically significant predictors in the adjusted model.

**Comment 2**: The findings summary in the Abstract should reflect adjusted results only. **Response:** We agree and have revised the Abstract to ensure that the interpretation fully reflects the multivariate findings, emphasising only education and full-time employment as independent predictors.

**Introduction**

**Comment 3**: The authors should include global context on PA prevalence among young adults in Asia, Europe, and Africa with citations.

**Response:** Thank you for this valuable recommendation. We have added a new paragraph providing global and regional comparative data on physical activity in youth populations from Asia, Europe, and Africa, supported by relevant references, to strengthen the study rationale and context.

**Methods**

**Comment 4:** The geographical coordinates should be removed, as they are not relevant to readers.

**Response:** We agree and have removed these details from the Study Setting section to enhance precision and readability.

**Comment 5**: The study setting description should be shortened as it contained excessive socio-economic detail.

**Response:** We have substantially condensed this section, retaining only the most relevant contextual information necessary to support the study’s objectives.

**Comment 6:** A sample size inconsistency was noted between the Study Population and Abstract.

**Response**: Thank you for highlighting this. We now clearly state that 1 063 participants were initially recruited, but only 762 participants with complete physical activity data were included in the final analysis. This clarification is reflected consistently in the Abstract, Methods, Results, and in all table captions.

**Comment 7**: The Data Collection section should be expanded to indicate whether the questionnaire was translated, interviewer training, and whether interviews were conducted face-to-face.

**Response**: This section has been revised to describe translation to Sepedi, back-translation procedures, structured interviewer training, and the face-to-face administration process using a standardised questionnaire adapted from the GPAQ.

**Comment 8**: The PA outcome definition should be clarified, including how ≥150 min/week was derived and whether multiple activity domains were included. **Response**: We have revised the Outcome Variable subsection to clearly indicate that physical activity minutes were derived using GPAQ scoring methodology across work, transport, and leisure domains, with WHO ≥150 min/week thresholds applied.

**Comment 9**: Ethics approval from the University of Limpopo should be explicitly stated. Response: We have updated the Ethical Considerations section to include the full ethics approval details from the Turfloop Research Ethics Committee (TREC/323/2017: IR-Renewed) along with a strengthened description of informed consent and confidentiality procedures.

**Results**

**Comment 10**: Sample size (N) should be consistently included in all tables.

**Response:** We appreciate this observation and have updated all tables and their captions to reflect a consistent sample size of N = 762.

**Discussion**

**Comment 11:** The key findings in the first paragraph should only highlight significant predictors and accurately reflect PA prevalence.

**Response:** The first Discussion paragraph has been rewritten to emphasise weekly PA as the most common pattern, high inactivity levels, and that only education and full-time employment were significant independent predictors. All interpretations now align with the adjusted model findings.

**Comment 12**: Statements regarding sex differences in PA should be revised as they were not statistically significant.

**Response:** Thank you for this correction. We have removed previous implications of significant gender disparities and now state clearly that sex was not independently associated with PA after adjustment, with supporting literature added.

**Comment 13**: Remove or revise statements relating income to PA since income was not significant in the adjusted model.

**Response:** We agree and have revised the text to clarify that while income showed associations in univariate analysis, it did not remain significant in multivariate models. We also contextualised that income effects may be mediated through education and employment.
